# Smart Vest for Respiratory Rate Monitoring of COPD Patients Based on Non-Contact Capacitive Sensing

**DOI:** 10.3390/s18072144

**Published:** 2018-07-03

**Authors:** David Naranjo-Hernández, Alejandro Talaminos-Barroso, Javier Reina-Tosina, Laura M. Roa, Gerardo Barbarov-Rostan, Pilar Cejudo-Ramos, Eduardo Márquez-Martín, Francisco Ortega-Ruiz

**Affiliations:** 1 Biomedical Engineering Group, University of Seville, 41092 Seville, Spain; talaminos@gmail.com (A.T.-B.); jreina@us.es (J.R.-T.); lroa@us.es (L.M.R.); gbarbarov@gmail.com (G.B.-R.); 2 Medical-Surgical Unit of Respiratory Diseases, University Hospital Virgen del Rocío, 41013 Seville, Spain; mariap.cejudo.sspa@juntadeandalucia.es (P.C.-R.); eduardomarquezmartin@neumosur.net (E.M.-M.); francisco.ortega.sspa@juntadeandalucia.es (F.O.-R.)

**Keywords:** Chronic Obstructive Pulmonary Disease (COPD), respiratory rehabilitation, respiratory rate, capacitive sensing, Internet of Medical Things (IoMT)

## Abstract

In this paper, a first approach to the design of a portable device for non-contact monitoring of respiratory rate by capacitive sensing is presented. The sensing system is integrated into a smart vest for an untethered, low-cost and comfortable breathing monitoring of Chronic Obstructive Pulmonary Disease (COPD) patients during the rest period between respiratory rehabilitation exercises at home. To provide an extensible solution to the remote monitoring using this sensor and other devices, the design and preliminary development of an e-Health platform based on the Internet of Medical Things (IoMT) paradigm is also presented. In order to validate the proposed solution, two quasi-experimental studies have been developed, comparing the estimations with respect to the golden standard. In a first study with healthy subjects, the mean value of the respiratory rate error, the standard deviation of the error and the correlation coefficient were 0.01 breaths per minute (bpm), 0.97 bpm and 0.995 (*p* < 0.00001), respectively. In a second study with COPD patients, the values were −0.14 bpm, 0.28 bpm and 0.9988 (*p* < 0.0000001), respectively. The results for the rest period show the technical and functional feasibility of the prototype and serve as a preliminary validation of the device for respiratory rate monitoring of patients with COPD.

## 1. Introduction

Chronic Obstructive Pulmonary Disease (COPD) was quantified as the sixth leading cause of death in 1990 and is projected to be the fourth in 2030 [1], also presenting secondary systemic manifestations [2]. Among these, the skeletal muscle dysfunction has a significant clinical importance because it contributes to poor health, increased healthcare utilization and even mortality [3]. The practice of individualized exercise has possibly been the most effective non-pharmacological intervention to improve the physical capacity, symptoms and health status in patients with COPD [4] and a reduction in healthcare utilization [5]. The difficulty of access for some patients to the training programs in ambulatory care [6] can be solved through supervised training programs at home [7], which can also serve as reinforcement in the long-term adherence of the patients.

e-Health systems, together with smart sensor devices that allow real-time monitoring of relevant clinical parameters at home, are considered a promising approach to the prevention and treatment of respiratory diseases [8]. The purpose of these systems is not only the telemonitoring and recording of physiological variables (respiratory and cardiac frequency, blood pressure, electrocardiogram, blood oxygen saturation [9], expired air flow, etc.), but also to motivate patients to adopt self-care skills, improve their quality of life, minimize recurrent exacerbations and hospital re-entry rates [10], as well as the safeguarding of patient safety and the establishment of an alarm system to address unwanted situations.

Although the respiratory rate is a vital sign of special importance in the context of the monitoring and follow-up of respiratory diseases, especially to avoid dangerous situations in critically-ill patients, it is still considered as the most neglected vital sign [11]. However, it could indicate a variety of pathological conditions in respiratory diseases, like COPD or sleep apnea, but also in cardiovascular and metabolic disorders [12]. The respiratory rate has been used to anticipate dangerous events such as cardiac arrest [13], to classify patients in intensive care units [14] and to predict complications or exacerbations in patients with cardiopulmonary diseases [15]. It is an essential parameter for the monitoring of postoperative patients [16] and for the detection of apnea or hypopnea events in pathologies related to sleep disorders.

Spirometry and capnography are the common techniques used for the monitoring of respiratory rate [17,18], but they represent an uncomfortable experience for the subject, disturbing the natural breathing; hence, they are not suitable for long-term application.

For continuous respiratory monitoring, numerous sensorization technologies have been proposed: The processing of information captured by an accelerometer can be used to derive the respiratory rate from the movements of the rib cage [19]. Spire [20] and MonBaby [21], the latter designed for babies, are examples of commercial devices based on accelerometers. Another device, Hexoskin [22], is based on respiratory inductance plethysmography, by contrast. Pressure sensors can also be used to estimate the respiratory rate [23]. The variations in electrical capacity between two conductive wires integrated in an elastic garment is employed in [24] to obtain the respiratory pattern. Fiber Bragg Grating (FBG) has also been employed to estimate the respiratory rate [25]. The impedance pneumograph is an indirect measurement of respiration through the analysis of thoracic impedance change [26]. This technique is the basis of the Zephir device [27]. Other proposals are based on the use of radar technology [28], surface diaphragm Electromyography (EMGdi) [29], respiratory temperature recorded using thermistors next to the mouth/nose [30], ultrasonic transmitters [31] or Photoplethysmogram (PPG) [32]. The Forced Oscillation Technique (FOT) is a promising tool for assessing future exacerbations in patients with COPD [33].

However, many of the proposed developments have the drawback of special sensor placement location, many times uncomfortable, or require thorough signal processing that severely impacts battery lifetime [34]. In addition, they do not have an accuracy comparable to that obtained by the obstructive methods (spirometry and capnography), even in experiments at rest.

From the point of view of communications, the rapid adoption of the Internet of Things (IoT) paradigm in different technological ecosystems is also having a significant impact on health information services, particularly on e-Health systems. In this sense, IoT is a concept that refers to the interconnection through the Internet of computing devices embedded in everyday objects [35]. These objects are physical elements with communication capability and programmable logic, including mainly sensor devices and actuators [36]. In the field of e-Health, the Internet of Medical Things (IoMT) paradigm is managing the technological transition from the traditional centralized systems, where the patient is considered as a passive element, towards patient-centered ecosystems and highly mobile environments [37,38].

The ubiquitous nature of IoT [39] enables the distribution of data between all communication entities, regardless of the underlying network topology, geographic location, type of device used or technological platform deployed. Real-time communications support, Quality of Service (QoS) policies and the publisher/subscriber-based communication pattern [40] are some advantages of the IoT paradigm, which represents an added value in e-Health systems to overcome some technological barriers that have limited the deployment of these types of systems. The IoT facilitates the communication and cooperation between systems and the parametrization and filtering of data handled. This allows the establishment of thresholds on the different types of data considered, thus also simplifying the management of alarms.

Health management and monitoring platforms integrated with the IoT technologies paradigm can further increase the intelligence, flexibility, scalability and interoperability of these systems [41]. Several publications have appeared in recent years describing monitoring systems of biomedical parameters using IoT platforms and wearable devices. For example, in [42] is proposed an ECG remote monitoring system that is dedicated to non-technical users and is integrated in an IoT infrastructure. Other ECG monitoring applications based on IoT have been presented [43,44] for the automatic, computer-based and accurate recognition of arrhythmias. In general, there are recent works presenting a diversity of wearable devices for the collection of biomedical parameters in IoT platforms, including posture [45], respiratory rate [46], blood pressure [47], blood glucose level [48] and more [49]. On the other hand, patient monitoring in rural areas can also benefit from IoT technologies [50] regarding the issues when doctors are unable to visit rural clinics frequently. Finally, there are also more generic IoT-based monitoring platforms [51,52,53] for a complete set of wearables. Although the success of e-Health platforms based on IoT has witnessed possibilities and opportunities, there are some issues and challenges still to solve; for example, standardization of IoT into a comprehensive semantic framework, limitations of sensors, interoperability between different protocols or data security.

In this context, the hypothesis being tested in this work is that capacitive sensor technology can be used for accurate, simple and non-obstructive respiratory rate monitoring of COPD patients during the rest period between respiratory rehabilitation exercises. In the field of e-Health, monitoring is considered non-obstructive when it does not create discomfort or inconvenience to the user, and the measurements can be performed without the user knowing that he/she is being monitored [54]. The aim of this paper is to provide a description of a first approach to the design and implementation of a smart sensor device based on capacitive technology integrated into a smart vest for an untethered, low-cost and comfortable breathing monitoring. The main novelty of this proposal compared to the literature and commercial devices is the application of the principle of capacitive sensing with the rib cage acting as an inhomogeneous dielectric body between two conductive textile electrodes. The technique was evaluated preliminarily by [11]; however, in this case, the electric capacity was measured by a commercial capacitance meter. In the present work is proposed a simple and sensitive measurement method of the electric capacity based on the Colpitts oscillator and the use of the internal timer of the microcontroller that serves as the processing unit of the smart vest to estimate the oscillation frequency, related to the capacity.

In order to facilitate the transmission of data generated by the device, a first approach to the technological communication infrastructure based on the IoT paradigm is described. The requirements of the use case presented demand real-time communications, data filtering, lightweight protocols, security and access control. These needs are aligned with the characteristics of any IoT system and particularly with the MQTT protocol, which provides support to the encryption and definition of access control lists, being also an open standard, facilitating interoperability among other suppliers and protocols.

Two quasi-experimental studies, one of them with healthy subjects and the other one with COPD patients, have shown the validity of the sensing technology and the proposed device. The results have been compared with respect to other non-obstructive devices and systems, both commercial and proposed by other researchers, in terms of accuracy and autonomy. The manuscript is structured as follows: Section 2 describes the material and methods used in the work. Section 3 shows the design and implementation of the smart vest, the design and preliminary development of the IoMT platform and the validation results. Section 4 analyzes the main results, and Section 5 gives the conclusions.

## 2. Materials and Methods

Considering the diversity of methods and techniques required to address the different issues regarding the design of the smart best, these will be explained in different subsections.

### 2.1. Methodology for Respiratory Sensing

Taking into account the proposed hypotheses, the capacitive sensor technology has been used as the basis of the signal acquisition stage of a portable smart sensor device for the non-obstructive monitoring of the respiratory rate. This technology provides high sensitivity with the added advantage that it does not require direct contact with the medium to be measured [11,34,55,56,57]. This technique is based on the measurement of the capacitance existing between two metal plates (electrodes) together with the thoracic tissue acting as a dielectric material. The mechanical changes produced by breathing cause variations in the capacitance, which can be correlated with patterns related to the respiratory rate. This technique has been validated by using commercial capacitance meters [11,34], but some authors have proposed portable systems based on an LC oscillator (the letter L represents an inductor, and the letter C stands for a capacitor) to accurately measure the capacitance [11,56,57,58,59].

A prototype of a capacitive sensor device has been researched and implemented. The characteristics of capacitive technology have allowed the system to be integrated into a smart vest that can be comfortably worn by the subject to be monitored. The main novelty of the device is a simplified front-end scheme for the measurement of the oscillation frequency, which takes advantage of the timer/counter characteristics of the microcontroller that captures and processes the signal, avoiding the use of an analog-digital conversion and providing greater sensitivity to the estimation of the electric capacity. Other innovations are related to the characteristics of the electrodes, implemented in a nickel on copper-plated polyester fabric, and a method for reducing noise by feedback of the oscillatory signal in the mesh that surrounds the signal cables. The device is considered smart, since in addition to capacitive sensing, it performs information processing, and IoT compliance standardizes wireless communication capabilities. For the processing of the data, an algorithm of low computational load is proposed to estimate the respiratory rate based on variations in the oscillation frequency, which can be efficiently executed in real time in an embedded way within the device.

### 2.2. Methodology for the Communication Platform

A first approach to the technological infrastructure based on the IoMT paradigm is proposed in order to facilitate the transmission of data generated by the capacitive sensor device to all the actors involved in the communication, including patients, professional and technical users, as well as family/caregivers members.

The choice of an IoT protocol is always a difficult issue to address, mainly because it requires an in-depth analysis of the requirements of the use case. The authors of this paper recently presented an exhaustive work of the most widely-used IoT protocols [60], considering not only a theoretical point of view, but also in terms of several performance metrics in a benchmarking study. Among the protocols analyzed are Data Distribution Service (DDS), Message Queue Telemetry Transport (MQTT), Constrained Application Protocol (CoAP), Java Message Service (JMS), Advanced Message Queuing Protocol (AMQP) and Extensible Messaging and Presence Protocol (XMPP). All of them are standardized by internationally-recognized organizations, are technology-independent, interoperable between different suppliers, have free software implementations for a large number of programming languages and are under continuous evolution. However, CoAP, MQTT and XMPP are lighter protocols and therefore more suitable for integration into IoT devices and particularly in the use case presented in this paper. Although all three protocols are based on the publisher/subscriber pattern, CoAP has some limitations in this regard due to the use of an observer, which is a subscription mechanism with restrictions. The results of the benchmarking carried out by the authors showed the significant advantages of MQTT in terms of battery consumption, given the low CPU and network traffic generated with respect to the other protocols studied. However, the main advantage of MQTT is its simplicity, which allows it to be integrated into any device with a low technical effort. Taking into account this benchmarking study, as well as similar studies by other authors, the MQTT standard (ISO/IEC 20922:2016) is proposed as the most suitable protocol to be used in the communication scenario presented in this work. Although the work is focused on a specific use case, the method applied can be used as a reference for the design of monitoring platforms based on IoT scenarios. The proposed platform allows integrating multiple sensors of a diverse nature with little technical effort, and the quasi-decentralized architecture based on standards and open technologies allows a high scalability when the number of sensors increases.

At the technological level, MQTT requires a broker or intermediary system that is responsible for receiving messages from publishers and redirecting them to subscribers. It is possible to deploy multiple brokers to work cooperatively. In terms of quality of service policies, MQTT provides three possible options: fire and forget, guaranteed delivery and guaranteed and exclusive delivery; in addition, at the security level exists an extension of MQTT called Secure MQTT (SMQTT) [61], which provides encryption and secure delivery to multiple communication nodes. In general, with MQTT, it is possible to generate data (publication) or consume them (subscription) in just a few lines of code and with low memory consumption. This lightweight quality is in accordance with the constraints of low performance devices where computational and network resources are limited. Finally, MQTT uses a space name hierarchy [62], which allows the subscription to data and facilities the definition of alarms. In short, its design has been focused on Machine-to-Machine (M2M) communications following an architecture based on the publish/subscribe pattern. This protocol is ideal for resource-constrained devices like the wearable used in this work.

**Figure 1:**
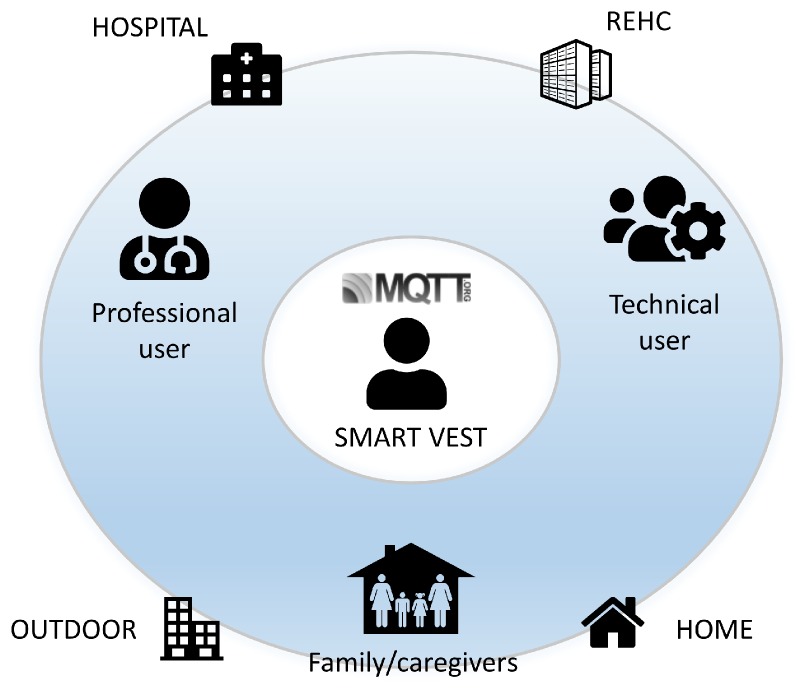
Diagram of the proposed IoMT platform.

The communication platform presented in this paper aims to provide support for the management of respiratory diseases for different services that are geographically dispersed. In particular, four actors have been identified (see Figure 1):

- Patient: This user wears the smart vest and is subject to continuous monitoring considering four possible scenarios (hospital, Remote E-Health Center (REHC), home and outdoor). The patient is the most active actor in communication and the main source of data generated throughout the platform.
- Professional user: receives relevant information on the activity of one or more patients, as well as notification of pre-established clinical alarms.
- Relative/caregiver: obtains information from a particular patient considering authorization mechanisms.
- Technical user: This user is located at the data center and receives a notification when there is any technical incident with the platform or the wearable device.

All the information managed in the monitoring scenario of patients undergoing respiratory rehabilitation [63] requires the use of alarms to detect possible anomalies in the controlled physiological variables and help to monitor the patient’s evolution. MQTT, as an IoT protocol, has been designed to achieve this purpose, i.e., to ensure the correct delivery of the data generated to all interested entities with data filtering conditions. Other features of the MQTT protocol also integrate additional functionalities that are aligned with the requirements of this work, including additional mechanisms to prioritize the messages, ensure the reception of data or provide data encryption and a robust access control system to restrict access to reading and writing operations.

Particularly, the smart vest has been designed for respiratory assessment (respiratory rate, inspiration time and expiration time) of COPD patients during the rest period between respiratory rehabilitation exercises. Technical alarms are triggered mainly when there is evidence that a patient’s smart vest has lost communication with the platform. Clinical alarms are related to respiratory rhythm abnormalities: triggered when the respiratory rate or inspiration/expiration times are outside certain configurable thresholds considered normal or theoretical for the patient after physical activity. These alarms are mainly received by professional users based on the thresholds set for each alarm, the level of authorization and the patient’s consent. On the other hand, the technical staff only receives technical alarms. Professional or technical users can customize both technical and clinical alarms for a single patient based on their needs and requirements. The adoption of IoT protocols enriches the platform to allow its future expansion by the integration of additional sensors.

### 2.3. Validation Methodology

Breathing monitoring was performed during the rest period between respiratory rehabilitation exercises emulating a remote monitoring at the patient’s home (application for which the device has been designed). Monitoring during the rest period between respiratory rehabilitation exercises addresses a wide range of respiratory rates while the patient recovers from the activity, which is desirable to allow a broad validation of operation [64].

As a first evaluation of the proposed technology, a use case has been selected for which respiratory monitoring in the resting state is of clinical relevance (respiratory rate monitoring of COPD patients during the rest period between respiratory rehabilitation exercises). Respiratory monitoring during two minutes at rest has been proposed as a method to evaluate respiratory rehabilitation efficacy [65] and the improvements in the breathing skills [66,67]. In this way, movement artifacts are avoided, providing greater veracity to the data in the evaluation process, both in the device under analysis as in the reference device [29,68]. This kind of monitoring can also be useful for the early detection of exacerbations [69] and safety monitoring during a program of home respiratory rehabilitation [70].

The operation of the device was contrasted with a portable and wireless clinical system designed for the performance of cardiopulmonary stress tests (Oxycon by CareFusion) based on a cycle ergometer, which was used as a reference standard. Its software, Oxycon Mobile, includes spirometry functions, flow and volume loops during the exercise, as well as the ability to analyze the respiratory rate and the inspiration and expiration times [71]. Both devices were placed on the volunteers (healthy subjects and patients with COPD) to perform simultaneous measurements. The inspiratory and expiratory times, as well as the respiratory rate were registered in both devices for further analysis. The reproducibility of the dynamic changes of the respiratory pattern was analyzed by comparing the results recorded by the sensor with those recorded in the ergometry.

To perform a comparative analysis of the capacitive device measurements with respect to the reference device, statistical parameters were used, such as the average value of the error and the Standard Deviation (SD) of the error. The Pearson correlation coefficient was also used, the results being considered statistically significant when p<0.05. In the case of the study with COPD patients, the agreement between the two devices was also evaluated using a Bland–Altman diagram, calculating the average difference between the 2 measurements and the corresponding SD. The Bland–Altman method also allows one to estimate the confidence intervals of the differences and estimate the accuracy of the result. The upper and lower concordance limits, defined by ±1.96 times the SD, are the limits of the 95% confidence interval in the measurement space. The MATLAB programming environment for Windows (Version R2016a, The MathWorks Inc., Natick, MA, USA) was used for data analysis.

The studies were approved by the Ethical Committee of the Virgen del Rocío University Hospital of Seville in Spain, and the participants signed informed consent.

With respect to the device performance analysis, power consumption was measured experimentally using an equivalent to the configuration proposed in [72]. In this case, a resistance of 2 ohms and an accuracy of 1% were placed on the power supply current. The voltage drop across the resistor was measured with an oscilloscope Agilent MSO6032A (Keysight Technologies, Santa Rosa, CA, USA), allowing the experimental characterization of the instantaneous current consumed by the device as a whole, or by a particular area such as the sensing unit.

## 3. Results

### 3.1. Design of the Capacitive Sensor Device

In the proposed paradigm, the sensor device is a capacitive smart sensor, which performs the acquisition and first processing of the sensory information for the real-time monitoring of the respiratory rate. The smart sensor has been conceived of to be integrated with an adjustable elastic garment on the chest of the user (smart vest). It communicates with a second device with more computational resources, referred to as the Decision-Analysis Device (DAD), thus establishing a Wireless Body Sensor Network (WBSN).

**Figure 2:**
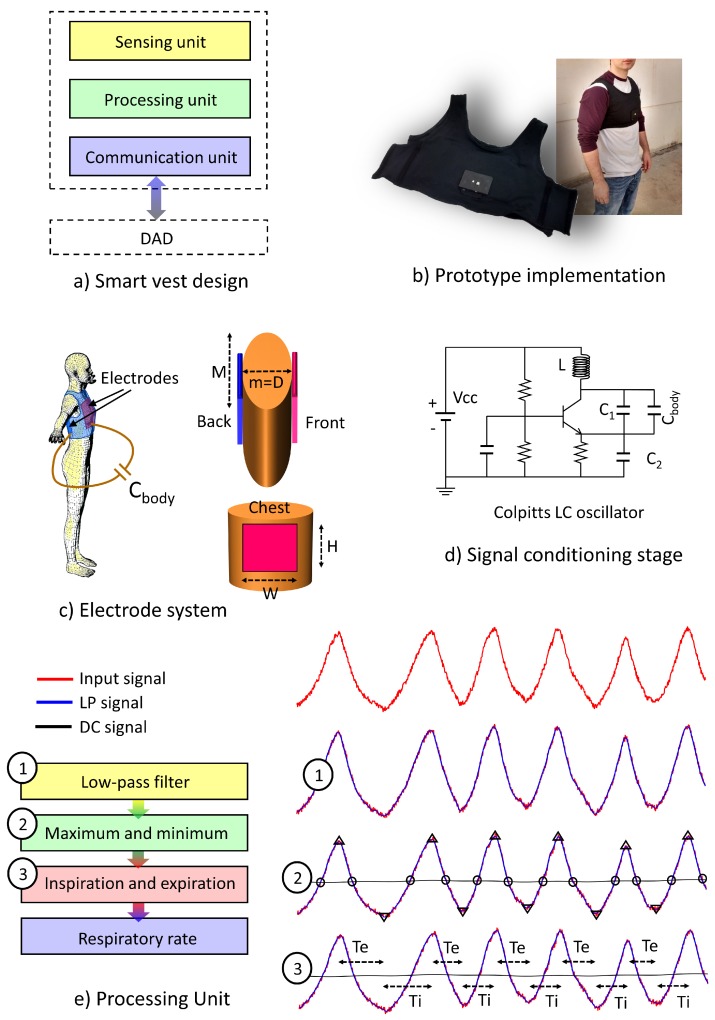
Smart vest: (**a**) design, (**b**) prototype implementation, (**c**) electrode system, (**d**) signal conditioning stage, (**e**) processing unit.

The design of the smart sensor device follows a modular and generic scheme supported by the following elements (see Figure 2):

- Sensing unit: This constitutes the acquisition element of the monitored physiological signal. It is formed by the electrode system and the signal conditioning stage.
- Processing unit: This module is responsible for transducing and processing the sensing signal to estimate the instantaneous respiratory rate. A PIC32 MX440F256 microcontroller microchip has been used for the purpose of data processing. Data can be stored in the internal memory or sent wirelessly in real time to the DAD. Table 1 shows a summary of the main characteristics of the processing unit [73]:
- Communications unit: This is responsible for the wireless transmission to the DAD of the respiratory rate and other related signals. The Bluetooth module HC-06 of Wavesen was employed to develop the communications. This transceiver was selected taking into account a trade-off between low cost, small size and easy configuration. The connectivity between the devices is implemented through the Serial Profile Protocol of the Bluetooth standard (SPP), with the sensor device functioning as the slave and the DAD as the master. An on/off communication scheme can be configured by the processing unit to reduce power consumption. In a debug communication mode, all data, sensing data (oscillation frequency, as will be seen later) and processing results (respiratory rate, inspiration time and expiration time) are sent continuously every time they are generated. In the normal communication mode, only processing results are sent with a latency period. The resulting data are saved until the end of the latency period, which can be wirelessly configured by means of a command and finally sent in packages.

The DAD is implemented through a mobile application for the Android operating system. It performs a deeper processing for a more thorough analysis of the sensory information and provides functionality/compatibility/support/compliance with IoMT for the communication with the REHC, if necessary.

### 3.2. Electrode System

During breathing, the changes in lung volume and the corresponding diaphragm movement result in the expansion-contraction of the chest and abdomen. The human body is mainly composed of a conductive electrolytic solution of water (relative permittivity, $\epsilon_{r}=80$) [11]. The air ($\epsilon_{r}=1$) that flows in and out of the body changes the permittivity of the chest, as well.

In the proposed system, two metal electrodes are arranged and face each other on the subject’s chest without being in direct contact with it (see Figure 2). The electrodes are implemented with an anti-corrosion-treated nickel on copper-plated polyester fabric (3M CN-3190 SHEET), 0.11 mm in thickness. Both electrodes have a rectangular shape, with a 25-cm width (*W*) and 13-cm height (*H*). They are integrated with an elastic garment (smart vest) in custom pockets that fits around the thorax, avoiding direct contact with the skin. The first electrode is located on the front of the chest on the sternum and the second electrode in a position parallel to the first one on the back. The position and size of the electrodes were fixed by an iterative procedure that maximized the variations of the sensing signal in forced maximum and minimum breathing experiments. In a first approximation in two dimensions, the cross-section of the chest can be approximated by an ellipse [74,75]. Taking as a reference the chest dimensions of the two healthy volunteers in the initial evaluation study, the average length of the major axis (*M*) of the ellipse corresponds to 37 cm and the average length of the minor axis (m) to 27.5 cm. Since the length of the major axis of the ellipse (*M*) is greater than the width of the electrodes (*W*), it can be considered that the deformation suffered in the electrodes when placed on the chest is small so that they can be approximated as a planar surface. In this case, the capacitance formed by the electrodes can be approximated by the capacitance between two parallel plates, which corresponds to the following expression [11]:

(1) $$C_{body}=\epsilon_{r,chest}\cdot \epsilon_{0}\cdot \frac{A}{D}$$

where $\epsilon_{0}$ is the permittivity of a vacuum, $\epsilon_{r,chest}$ is the global relative permittivity of the chest, *A* is the area of the electrodes (0.0325 m^2^) and *D* is the distance between the electrodes (0.275 m if the average length of the minor axis of the ellipse is considered). During respiration, a cyclic movement of expansion/contraction of the rib cage occurs. In the case of the study carried out on young volunteers, this effect corresponded to an average increase of 2 cm in the perimeter of the chest section during normal breathing, on a base perimeter of 104 cm at the time of expiration. This average measurement is very close to the perimeter of the ellipse estimated by Ramanujan’s approximation [76], 102 cm, which allows validating the hypothesis of approaching the cross-section of the chest as an ellipse. In addition, if it is considered that the chest expands equally in all directions, and also taking into account Ramanujan’s approximation, the increase of 2 cm in the perimeter would be associated with an increase of 0.5 cm in the distance Dbetween electrodes at the time of inspiration. This increase in the distance between electrodes ΔD would be in turn associated with a decrease in base capacitance $\Delta C_{body}$ according to the following expression [77]:

(2) $$\Delta C_{body}=-\frac{C_{body}}{D}\cdot \Delta D$$

As a consequence, it can be concluded that the volume changes when breathing will result in a variation in capacitance, so that at the instant of maximum lung volume (maximum *D*), the capacitance is minimal and the instant of minimum lung volume (minimum *D*) corresponds to the maximum capacitance.

Although the geometric data analyzed correspond to healthy subjects, the considerations can also be extended for the case of patients with COPD. A study of these patients [74] showed a mean perimeter close to 104 cm at the time of inspiration and 102 cm at the time of expiration (with a standard deviation close to 10 cm), which also implies a variation of the chest perimeter close to 2 cm.

### 3.3. Signal Conditioning Stage

The chest capacitance can be accurately measured by an LC oscillator. LC oscillators are circuits formed by a shunt coil and capacitor (tank LC circuit). Keeping oscillating stability in an oscillator is not a simple task [78]; however, the Colpitts oscillator presents a bifurcation phenomenon that makes it robust in its operation [79]. For this reason, a common-base configuration of the Colpitts oscillator has been used for the design of the device. Figure 2 also shows a schematic drawing of the oscillator. Its operation is based on the exchange of energy between the capacitor, which stores energy in the form of electric charge, and a coil, in the form of a magnetic field. This exchange of energy generates a sine wave, which is maintained in time thanks to a bipolar transistor, which performs a feedback and amplification of the wave. The oscillation frequency $f_{O}$ directly depends on the inductance value of the coil and the electrical capacities according to the following equation:

(3) $$f_{O}≃\frac{1}{2\pi \sqrt{L\frac{C_{2}\cdot C_{body}}{C_{2}+C_{body}}}}$$

It is not considered an exact value for the expression since it implies a simplification according to which the value of the capacitance $C_{1}$, set to 10 pF, has a value much lower than $C_{body}$, so it can be neglected in the formulation of the LC oscillator with two capacitances in series [58]. However, $C_{1}$ is included in the design to ensure oscillation in situations where $C_{body}$ is very low, making the design more robust, since for the device to work, it is necessary that there is oscillation.

The value of the inductance *L* was 330 µH, and the value of the capacitance $C_{2}$ was 100 nF. The parameters of the oscillator were established by electronic simulation (Orcad Capture v16.0), maximizing a sensitivity parameter related to the absolute variation of the oscillation frequency caused by variations in the capacitance $C_{body}$ of 50 pF.

In the experiments, the oscillation frequency was in the range from 400 kHz–650 kHz, corresponding to a capacitance $C_{body}$ range from 484 pF–182 pF, respectively. The lowest capacitance value is 18-times higher than $C_{1}$, allowing one to verify the simplification made in (3).

The electrodes are attached to the oscillator by means of coaxial cables. The output voltage of the oscillator, decoupled by an operational amplifier in the voltage follower configuration, is applied to the metal mesh of the cables, since previous experiments showed that this scheme reduced the noise and increased the sensitivity. Finally, an operational amplifier in the comparator configuration converts the sine wave into a square wave.

The comparator allows the oscillator to be decoupled from the processing unit, since it has been verified that with a direct connection, the input impedance of the microcontroller sensitively affects the oscillation frequency. This modular design allows the sensing unit to be independent of the device that uses it. In addition, the method of detecting the oscillatory signal based on the measurement of the number of edges, which is explained in detail at the beginning of the next section, works more robustly with a square signal, avoiding losses in the capture of edges if the quality of the oscillatory signal decreases.

### 3.4. Processing Unit

Against most of the sensing devices that analyze biomedical parameters, the smart vest does not use an Analog/Digital Converter (ADC) module, but a frequency-to-signal conversion scheme. To simplify the necessary hardware resources for the measurements, the microcontroller is configured so that an internal timer/counter ($T_{1}$) of 16-bit works as an external input source, with the oscillatory signal generated by the sensing unit. The counter is incremented with each rising edge of the oscillatory signal, and in an auxiliary register, the number of times the counter overflows during the sampling period is registered. A second timer/counter ($T_{2}$) of the processing unit is responsible for managing the sampling frequency of the device, generating an interruption each time this counter overflows (50 milliseconds is the sampling frequency established in the experiments; other values can be configured). At that moment, the data of the register and the value of $T_{1}$ are captured and processed to determine the oscillation frequency, which corresponds to 20-times the number of rising edges of the signal during the period between samples. Just after the data capture, $T_{1}$ and the register are reset for a new measurement cycle to provide more accurate estimations.

The total number of respiration cycles per minute is called the respiratory rate. Other parameters such as inspiration time and expiration time are also computed by the device, which provide more accurate information on the patient’s breathing pattern. To perform the estimation of these parameters, the instantaneous oscillation frequency is processed by following a four-step algorithm adapted from [57] (see Figure 2):

1. In the first step, the signal related to the instantaneous oscillation frequency is filtered by a low-pass fourth-order Butterworth filter with a cutoff frequency of 4 Hz, to smooth the signal and remove noise components (LPsignal in Figure 2). The cutoff frequency was selected through an iterative process to eliminate the noise components of high frequency from the signal associated with the oscillation frequency, but maintaining the characteristics of the respiratory pattern to accurately detect the duration of inspiration and expiration times.
2. In the second step, the temporal instants corresponding to the maximum and minimum values of the LP signal are calculated. For this purpose, a second signal characteristic of the low frequency variation of the input signal was obtained through a low-pass second-order Butterworth filter with a cutoff frequency of 0.05 Hz (DC signal in Figure 2). In the same way, the cutoff frequency was selected through an iterative process so that the DC signal can adapt to the drifts of the input signal, but with enough flexibility to respond to changes in the operating point of the oscillator. The cut points of the LP signal with the DC signal are used to define the search intervals of the maximum and minimum values of the LP signal, thus avoiding possible local maximums and minima caused by instabilities of the respiratory pattern.
3. In the third step, the time instants of inspiration and expiration are determined taking into account that the instant of maximum lung volume corresponds to a maximum in the oscillation frequency and the instant of minimum lung volume corresponds to a minimum in the oscillation frequency. The expiration time (Te) is calculated as the time elapsed between a successive maximum and minimum in the LP signal, while the inspiration time (Ti) corresponds to the time between a successive minimum and maximum.
4. In the fourth step, the instantaneous respiratory rate is estimated as the inverse of the full respiratory cycle (inspiration time plus expiration time) and averaged over a configurable period of time.

### 3.5. Design of the IoMT Platform

The heterogeneity of scenarios and actors involved in the platform proposed in this work is in accordance with a many-to-many communication model where all nodes can generate and receive data at the same time. However, proper security mechanisms must be implemented to provide confidentiality, authentication and access authorization. From this perspective, the MQTT protocol provides technical features that cover all these aspects. In addition, it also offers mechanisms in terms of scalability, so that the increase in the number of elements does not have a significant impact on the performance of the platform.

Figure 1 shows a simple diagram of the different scenarios and stakeholders involved in the platform, with the patient as the main focus of all the communications. In general, the other actors play a passive role, receiving information from the patient based on their needs, their level of authorization and the patient’s consent.

On a more technical level, the DAD (which will become the master of a piconet network) broadcasts messages to discover each patient’s smart vest (slaves). In this phase of communication (paging), devices exchange data such as the physical address, and the master initiates the connection. Once the communication is established, the DAD can send data to any of the patient’s smart vests and request data from them, as well, but one patient’s smart vest can only be connected to an intermediary element. As mentioned in Section 3.1, DAD is implemented by using a smartphone, and a mobile application has been developed for the Android operating system in the context of IoMT, where family members, patients, professional and technical users view information generated for device sensors in real time. This communication remains persistent while the patient follows the rehabilitation exercise plan, and at the same time, the DAD locally stores the data monitored by the smart vest. If an alarm is triggered during the monitoring, the DAD notifies the event via the MQTT protocol. If the DAD does not have connectivity at that time (for example, certain cases of patients on public roads), the alarms will be recorded in an off-line buffer waiting for the network to recover. At the other extreme (REHC), the technical user will be informed that communication with the patient has been lost. Other information not associated with alarms is collected and transmitted from the DAD to the platform via the 4G network.

**Figure 3:**
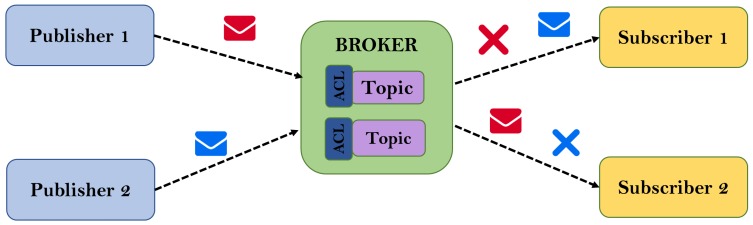
Generic operation scheme of the MQTT protocol.

Particularizing the MQTT protocol and following the publish/subscribe pattern, data sent by the DAD are published on different topics depending on the type of data and the type of alarm. The different stakeholders interested in data or alarm events have a unique identifier and an associated Access Control List (ACL). On the other hand, the data model is constituted under a hierarchical structure, according to best practices for the MQTT protocol, and the subscription by content filtering is covered by wildcards [80]. A generic scheme of the technological components and data flow of the MQTT protocol is shown in Figure 3.

### 3.6. First Evaluation Results

**Figure 4:**
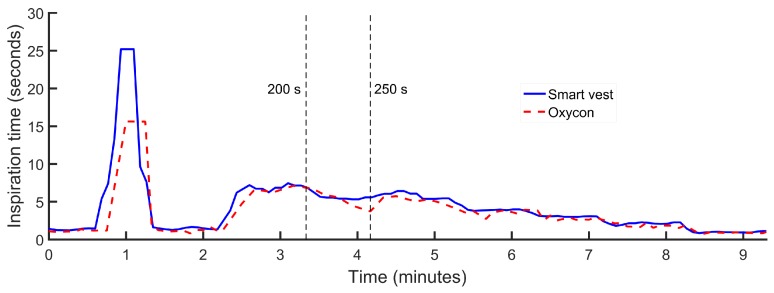
Inspiration time in one of the experiments with healthy subjects.

**Figure 5:**
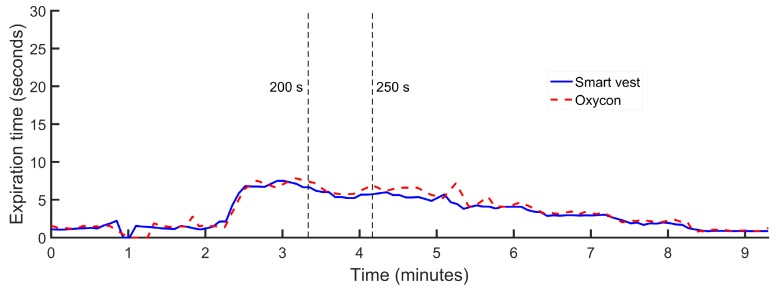
Expiration time in one of the experiments with healthy subjects.

**Figure 6:**
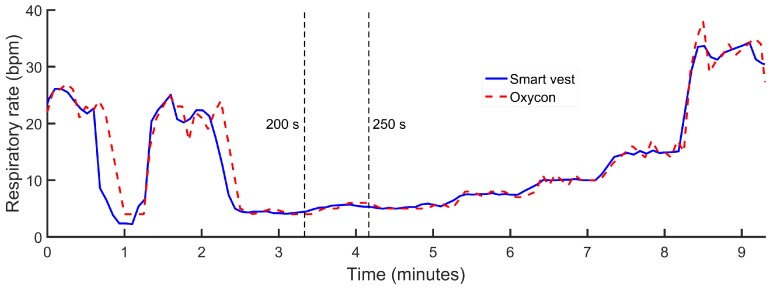
Respiratory rate in one of the experiments with healthy subjects.

**Figure 7:**
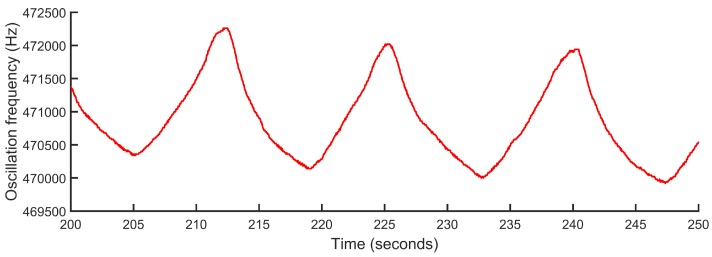
Example of oscillation frequency $f_{O}$ variation in one of the experiments with healthy subjects.

The initial evaluation on healthy subjects was carried out on two male volunteers of 36 and 42 years, 180 cm and 172 cm in height and 100 kg and 80 kg in weight, respectively. During the experiments, the volunteers performed a programmed sequence of activities. In the first phase of the experiments, the volunteer remained seated. In the second phase, the subject remained standing and was increasing the inspiration and expiration times, sequentially and in a controlled way. This experiment was repeated twice by each of the volunteers. The average duration of the experiments was 7 min and 30 s, and as the volunteers performed the experiment twice, the data recorded together corresponded to a total time of 30 min of measurement. As an example of the data obtained in the experiments, Figure 4, Figure 5 and Figure 6 show the inspiration time, expiration time and respiratory rate obtained in one of the experiments conducted by one of the volunteers, approximately 9 min in this case. At a certain moment at the beginning of each experiment, apnea was performed for 25 s, as shown by the maximum time of inspiration. Figure 7 shows an example of a signal generated by the signal conditioning stage. This signal represents the variations of the oscillation frequency of the LC oscillator as a consequence of the respiration process. The processing result of this oscillatory signal is shown as a clarification in the time interval delimited by dashed lines in Figure 4, Figure 5 and Figure 6.

Table 2 shows the comparative analysis results of the capacitive device measurements with respect to the reference device in the initial evaluation on healthy subjects (mean value of the error, SD of the error, correlation coefficient, *p*-value) for the inspiration and expiration time (in seconds) and respiratory rate (in breaths per minute or bpm).

In this first evaluation, the mean error of the inspiration time estimation was positive, indicating that this parameter was overestimated by the sensor. On the other hand, the mean error in the expiration time was negative, which allows us to conclude that this parameter was underestimated in these experiments. Although these differences were compensated for each other in the estimation of the mean value of the respiratory rate, the standard deviation shows a cumulative effect of the errors.

### 3.7. Second Evaluation Results

Nine patients with COPD included in a respiratory rehabilitation program participated in the second evaluation study. Table 3 shows the anthropometric characteristics of the volunteers participating in the study. In order to promote a greater variation in the respiratory rate and a larger range of evaluated values, the recording of the respiratory signals was simultaneously performed by the capacitive and the reference devices during a pause of approximately two minutes between exercises of the rehabilitation program. As already mentioned in Section 1, this kind of monitoring has been proposed as a method to evaluate respiratory rehabilitation efficacy [65] and the improvements in breathing skills [66,67].

**Figure 8:**
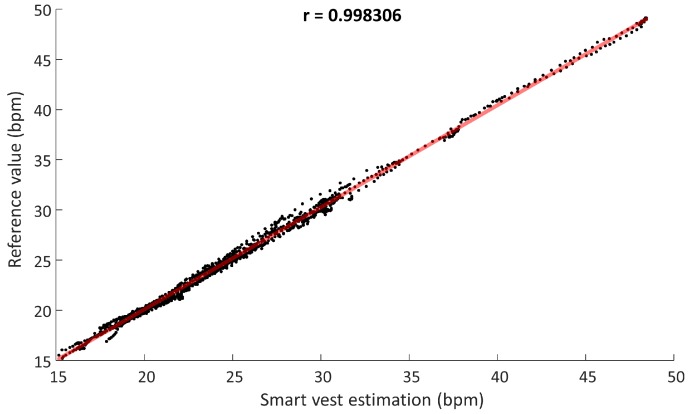
Measurement correlation graph in the study with COPD patients.

Figure 8 shows the linear correlation obtained in the measurements of both devices in bpm. A correlation coefficient r=0.9988 (p<0.0000001) was reached by the least squares method.

**Figure 9:**
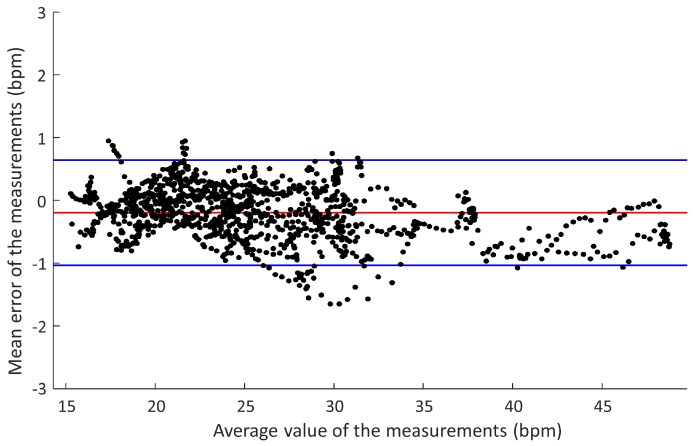
Bland–Altman diagram of the capacitive device measurements with respect to the reference in the study with COPD patients.

Figure 9 represents the Bland–Altman diagram of these measurements, with an average error of −0.14 bpm (of the capacitive sensor device with respect to the reference) and a standard deviation of 0.28 bpm. The lower limit of the 95% confidence interval was −0.68 bpm, and the upper limit was 0.4 bpm.

### 3.8. Smart Vest Performance

The autonomy of the device is a parameter of significant importance for the usability of the smart vest. It should be extended as much as possible to avoid inconveniences to users (monitored and professional) with recharges or battery replacements, which would negatively affect the usability of the system and cost. Table 4 shows an average estimation of the power consumption of the device as a whole in two different situations: debug communication mode and normal communication mode with a 1-min latency period. Considering that the battery of the device had a capacity of 2000 mAh, the autonomy of the smart vest is estimated in both situations. Power consumption was estimated taking into account that the supply voltage was 3.3 V. In addition, the current consumption corresponding only to the sensing unit is also shown in the table.

The electronic circuit of the smart vest has an area of 11 cm × 8 cm (width × height) and is located ahead of the front electrode, without affecting the body capacitance. Its weight is 50 g. The smart vest as a whole has a width of 36 cm and a height of 19 cm. These dimensions can be adjusted to the characteristics of the person thanks to the elastic characteristics of the garment. Its overall weight is 250 g.

The algorithm was embedded in the physical processing unit of the smart sensor device. The algorithm code occupied 1360 bytes of the program memory and 756 bytes of the data memory.

### 3.9. Sensitivity Analysis

An advantage of the measurement method used for the sensor is its high sensitivity. To analyze this aspect, three operating points will be considered in the range of oscillation frequencies obtained in the experiments: the minimum frequency of 400 kHz, the intermediate frequency of 525 kHz and the maximum frequency of 650 kHz, which correspond to a $C_{body}$ of 484 pF, 279 pF and 182 pF, respectively. The sensitivity of the sensor is established as the minimum quantity measurable by the device. Table 5 shows the results of the analysis at the three operation points. The first row shows the maximum number of detectable edges of the oscillatory signal during the period between samples calculated as the division of the sampling period between the period of the oscillation frequency. The data in the second row correspond to the oscillation frequencies associated with the hypothetical cases in which the number of edges was reduced by one unit (reduced frequency, associated with the minimum detectable difference of frequencies). The third row indicates the sensitivity in the oscillation frequency, calculated as the difference between the current oscillation frequency and the reduced frequency. The fourth row shows the sensitivity in the measurement of the capacitance $C_{body}$, as the difference of capacitances obtained from (3) for the current oscillation frequency and the reduced frequency. The last row indicates the sensitivity in the measurement of the distance between electrodes *D*, calculated from (2) and considering the average distance between the electrodes *D* of 0.275 m. Other different sensitivities can be obtained using other sampling frequencies.

## 4. Discussion

The present work has shown the description of a first approach to the design of a smart sensor device for the monitoring of the respiratory rate. It has also shown a first validation of the device on the basis of two quasi-experimental studies, one of them with healthy subjects and the other with COPD patients. These studies were conducted in a controlled clinical environment, using a precision clinical system as a reference method.

The results obtained in the study with healthy subjects for the comparative analysis of respiratory parameters (inspiratory time, expiratory time and respiratory rate), estimated by the capacitive device with respect to the reference device, have shown the technical and functional feasibility of the proposed prototype. Furthermore, the proposed sensor has shown better performance with respect to the reference system in some conditions of this study. Finally, the results obtained in the second evaluation study have served to show a preliminary validation of the device in the monitoring of the respiratory rate of patients with COPD. The errors assumed by the 95% confidence interval shown in the Bland–Altman diagram of Figure 9 are considered clinically acceptable. The number of measurements outside the confidence interval is very small, and their values are very close to the limits.

According to the results obtained in the first evaluation, the smart sensor was more precise at estimating the inspiratory and expiratory times. As can be seen in Figure 4, the duration of the period during which the volunteers held their breath (inspiration) was approximated accurately by the smart sensor device (25 s). On the other hand, the average made by the reference device does not allow one to reach this precision.

Another aspect that stands out from the results is the stability of the estimations. In the second phase of the experiments, the volunteers increased the respiratory rate incrementally, controlling the inspiratory and expiratory times in a programmed schedule. As can be seen in Figure 5 and in the second phase of the experiments, the signal estimated by the proposed sensor approximates the predicted stepped signal with greater stability and a smaller number of artifacts than the reference device.

As an added value with respect to the reference device, the proposed system offers a real-time estimation of the respiratory pattern waveform at each moment, as shown by the signal in Figure 7. The waveform provides more detailed information of the respiratory process and its intensity, which can be useful in the diagnosis and monitoring of multiple pathologies.

In addition, the smart vest was much more comfortable for the subject than the reference device, which captures the respiratory rate through a mask that includes a spirometer. As the respiratory rate is also influenced by the autonomic nervous system, the feeling of confinement and discomfort resulting from the signal capture system of the reference pattern could involuntarily affect the respiratory response, a circumstance that would prevent the proposed sensor. Moreover, the low cost of materials employed for the development of the smart capacitive device must be highlighted.

To perform a comparative analysis of the capacitive device measurements with respect to the reference device, statistical parameters were used, such as the average value of the error and the SD of the error. The Pearson correlation coefficient was also used, considering results statistically significant when p<0.05. In the case of the study with COPD patients, the agreement between the two devices was also evaluated using a Bland–Altman diagram, calculating the average difference between the two measurements and the corresponding SD.

The Bland–Altman diagram results are usually employed to estimate the respiratory rate accuracy in validation studies of new devices against the reference pattern [81]. A comparison of the results obtained with the proposed smart vest is presented in Table 6 with respect to other devices and systems recently proposed by other authors, including evaluations regarding commercial devices. The table shows the mean value of the error committed by different devices against the reference standard, as well as the limits of the 95% confidence interval in the measurement space, all of them in bpm. In the same table, the protocol used in the experimentation is shown. To allow comparison, all the studies analyzed evaluated the respiratory rate in rest situations. When a preliminary evaluation of a technology for respiratory monitoring is carried out, it is common to use experimentation at rest to minimize muscle artifacts that could interfere with measurements [29,32,68]. Additionally, the table also includes a description of the method used by the sensor device to transmit the data, if any, as well as the way in which the processing to estimate the respiratory parameters is performed.

In the experiments carried out, the proposed device was more accurate than the rest of the recent technologies compared in the table. Another merit of the proposed device is its wireless communication capability. Only a commercial device also had this capability. In addition, the data processing to estimate the respiratory rate is performed in real time inside the device by means of an embedded algorithm. Only two commercial technologies provided this benefit. This is achieved thanks in part to the simplicity and low computational load of the proposed algorithm, which also allows a lower need of energy consumption for data processing.

The device performance with respect to energy consumption is not easy to compare, since these data are not commonly presented in validation studies. Even so, some data are shown to allow a comparative analysis. The Hexoskin smart shirt has an autonomy between 12 and 30 h of battery life [22]. The device proposed in [64] has a current consumption between 1000 and 1500 mA and approximately 5.6 h of autonomy in continuous use. The autonomy of the Zephyr commercial device is 35 h [27]. However, the best features are provided by the system described in [68], which had a five-day battery life. The smart vest proposed in the present work has an autonomy of 45.5 h in debug communication mode and 68.9 h in normal communication mode (1-min latency), which surpasses almost all the technologies mentioned above. In addition, the low power consumption of the sensing unit, only 1.3 mW, highlights the benefits of the proposed capacitive sensing technology.

Despite the size of the sample analyzed in these first two evaluation studies, two healthy volunteers and nine patients suffering from COPD, the number of measurements has been high, with a total data capture time of approximately 50 min. Although the measurements were performed at rest, they were affected by multiple internal and external disturbances. We consider that the determinations have statistical significance at the sample level, but not in terms of the diversity of the individuals participating in the studies.

It should be also considered that the estimation of the respiratory parameters made by the sensor (inspiration time, expiration time and respiratory rate) are made based on a relative measure, and not absolute capacity. The measurement conditions of the sensor can vary for different people and even for the same person at different times (the vest is not always in the same position, parasitic capacitances, etc.), but the measurement method is robust when using a relative measurement. In this way, the effect of artifacts related to the conditions of measurement can be minimized.

Last but not least, a first approach to the design of a e-Health platform to support the smart vest communications has also been presented based on the IoMT paradigm. Good practices have been followed in its design considering the functional and performance analysis carried out in a previous work of the authors [60]. In general, in a simple communication using publish/subscribe pattern, performance is almost similar for all IoT protocols. However, MQTT presents some strengths [60], such as lightweight, low CPU and memory usage, low latency, compact binary packet payload and WebSocket support.

The adoption of MQTT for the use case proposed in this paper presents significant benefits given the need for personalization of the patient’s care plan, considering also the variability of data handled for the patient: personal information, anthropometric factors, life habits, symptoms, typology and severity of the pathology. Although there are many successful cases of MQTT in the field of e-Health, to the best of the author’s knowledge, this is the first paper to consider its application in a communications scenario focused on respiratory rehabilitation.

## 5. Conclusions

This work has shown a first approach to the design and implementation of a smart sensor device integrated with a smart vest for respiratory rate monitoring. Such a device can be employed in an application for breath monitoring of COPD patients during the rest period between respiratory rehabilitation exercises in their own home. The design requirements of such a device were low cost, unobtrusive and ease of use for the patient. To this end, the system utilizes non-intrusive technologies based on the capacitive sensing principles of low-cost LC oscillators with high detection sensitivity.

A first validation of the capacitive sensor has been performed through two quasi-experimental studies carried out on healthy subjects and COPD patients in a controlled clinical environment, using a precision clinical system as the reference measurement system. The precision features of the smart vest have been compared with other commercial devices and systems proposed by other authors, showing the validity of the proposal.

The interoperable connectivity of the device in the context of remote biomedical monitoring has been approached through the proposal of an IoMT framework based on the MQTT protocol. The architecture, based on standards and open technologies, can be used as a reference to aid e-Health developers to deploy IoT monitoring platforms, taking into account technical features such as security, interoperability and scalability when the number of sensors grows.

## Figures and Tables

**Table 1:** Processing unit characteristics.

Power Supply	Speed	Flash Memory (Program)	SDRAM Memory (Data)	Timer Counter
3.3 V	80 MHz	256 KB	32 KB	16-bit

**Table 2:** Statistical results of the comparative analysis in the initial evaluation of healthy subjects for the proposed sensor device with respect to the reference method.

	Mean Value of the Error	SD of the Error	Correlation Coefficient	*p* Value
Inspiration time	0.3 s	0.44 s	0.975	*p* < 0.001
Expiration time	−0.41 s	0.5 s	0.975	*p* < 0.001
Respiratory rate	0.01 bpm	0.97 bpm	0.995	*p* < 0.001

**Table 3:** Anthropometric characteristics of the COPD patients in the second evaluation study.

	Total	Men	Women	
**Number of Volunteers**	**9**	**6**	**3**	
	**Minimum Value**	**Average Value**	**Maximum Value**	**SD**
Weight (kg)	58	81.1	106	14.9
Age (years)	55	64	76	6.6
Height (cm)	152	167.6	185	10.3
Body Mass Index	18.7	29.2	39.3	6.3

**Table 4:** Power consumption features of the smart vest.

	Current Consumption (mA)	Power Consumption (mW)	Autonomy (Hours)
Debug communication mode	43.9	145	45.5
Normal communication mode	29	95.8	68.9
Only sensing unit	0.4	1.3	5166.5

**Table 5:** Results of the sensitivity analysis.

	400 kHz	525 kHz	650 kHz
Maximum number of detectable edges	20,000	26,250	32,500
Reduced frequency	399,980 Hz	524,980 Hz	649,980 Hz
Oscillation frequency sensitivity	20 Hz	20 Hz	20 Hz
Capacitance $C_{body}$ sensitivity	0.048 pF	0.021 pF	0.011 pF
Distance *D* sensitivity	27.6 µm	21 µm	17 µm

**Table 6:** Comparison of the respiratory rate results obtained with the smart vest regarding other studies (average error in bpm, limits of the 95% confidence interval (CI) in bpm, experimental protocol, method used for communication and method employed for processing).

Ref.	Method	Average Error	CI	Protocol	Communication	Processing
This	Capacitive	−0.14	−0.68–0.4	rest (after exercising)	Bluetooth	Embedded
[19]	Accelerometer	−0.21	−2.32–1.89	rest (sitting)	Wired	PC (MATLAB)
[28]	Radar	−0.14	−1.1–0.86	rest (lying)	Wired	PC
[82]	Inductance	1.6	−0.03–3.17	rest	Bluetooth 4.0	Embedded
[26]	Impedance	0.13	−1.5–1.7	rest (lying)	-	Embedded
[25]	FBG	−0.01	−2.1–2.1	rest (lying)	Wired	PC
[32]	PPG	0.21	−1.1–1.5	rest (lying)	Wired	PC (MATLAB)
[29]	EMGdi	0.01	−2.39–2.41	rest (sitting)	Wired	PC (MATLAB)

## Abbreviations

The following abbreviations are used in this manuscript:

ACL	Access Control List
ADC	Analog/Digital Converter
AMQP	Advanced Message Queuing Protocol
bpm	breaths per minute
CI	Confidence Interval
CoAP	Constrained Application Protocol
COPD	Chronic Obstructive Pulmonary Disease
DAD	Decision-Analysis Device
DDS	Data Distribution Service
EMGdi	Surface Diaphragm Electromyography
XMPP	Extensible Messaging and Presence Protocol
FBG	Fiber Bragg Grating
FOT	Forced Oscillation Technique
IoMT	Internet of Medical Things
IoT	Internet of Things
ISO	International Organization for Standardization
JMS	Java Message Service
M2M	Machine-to-Machine
MQTT	Message Queuing Telemetry Transport
WBSN	Wireless Body Sensor Network
PPG	Photoplethysmogram
QoS	Quality of Service
REHC	Remote e-Health Center
SD	Standard Deviation
SMQTT	Secure MQTT
SPP	Serial Profile Protocol
